# Role of Kisspeptin and Neurokinin B in Puberty in Female Non-Human Primates

**DOI:** 10.3389/fendo.2018.00148

**Published:** 2018-04-06

**Authors:** Ei Terasawa, James P. Garcia, Stephanie B. Seminara, Kim L. Keen

**Affiliations:** ^1^Wisconsin National Primate Research Center, University of Wisconsin, Madison, WI, United States; ^2^Department of Pediatrics, University of Wisconsin, Madison, WI, United States; ^3^Reproductive Endocrine Unit and the Harvard Reproductive Sciences Center, Department of Medicine, Massachusetts General Hospital, Boston, MA, United States

**Keywords:** kisspeptin, neurokinin B, GnRH, puberty, nonhuman primate

## Abstract

In human patients, loss-of-function mutations in the genes encoding kisspeptin (*KISS1*) and neurokinin B (*NKB)* and their receptors (*KISS1R* and *NK3R*, respectively) result in an abnormal timing of puberty or the absence of puberty. To understand the neuroendocrine mechanism of puberty, we investigated the contribution of kisspeptin and NKB signaling to the pubertal increase in GnRH release using rhesus monkeys as a model. Direct measurements of GnRH and kisspeptin in the median eminence of the hypothalamus with infusion of agonists and antagonists for kisspeptin and NKB reveal that kisspeptin and NKB signaling stimulate GnRH release independently or collaboratively by forming kisspeptin and NKB neuronal networks depending on the developmental age. For example, while in prepubertal females, kisspeptin and NKB signaling independently stimulate GnRH release, in pubertal females, the formation of a collaborative kisspeptin and NKB network further accelerates the pubertal increase in GnRH release. It is speculated that the collaborative mechanism between kisspeptin and NKB signaling to GnRH neurons is necessary for the complex reproductive function in females.

## Introduction

Puberty is a transitional period between the sexually immature juvenile stage and adulthood, after which full reproductive function is attained. In the 1980s, the concept that an increase in GnRH release initiates puberty was established. Although from 1980 to 2000, it became clear that central inhibition over GnRH release during the prepubertal period needs to be removed or diminished in primates (1), the discovery that gene mutations in kisspeptin (*KISS1*) and its receptor (*KISS1R*) in human patients result in delayed puberty or no puberty (2, 3) has generated great progress in understanding the mechanism of puberty. Together, with the subsequent findings showing that mutations in neurokinin B (NKB) and its receptor (*NK3R*) in humans also result in delayed puberty or no puberty (4), this led us to study how kisspeptin and NKB signaling changes before and after puberty onset in female rhesus monkeys. This short review article summarizes our findings and perspectives regarding the role of kisspeptin and NKB signaling in puberty onset in females.

## Developmental Changes in Gonadotropin Secretion in Female Rhesus Monkeys

**Figure 1:**
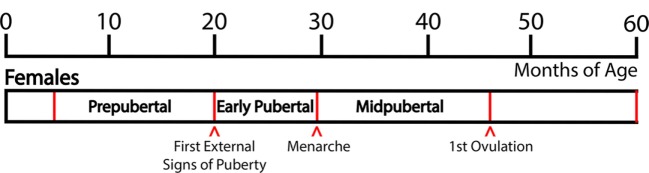
Developmental stages of pubertal progression in female rhesus monkeys. Based on changes in physiological characteristics and in circulating hormone levels during the developmental course, prepubertal, early pubertal, and midpubertal stages are defined as shown in this figure. Actual age of the onset of puberty and subsequent progress vary among animals.

Based on developmental changes in LH and FSH levels and external signs of puberty, we have defined the pubertal stages as follows: The “prepubertal stage” is when female monkeys do not exhibit any external signs of puberty and gonadotropin levels are low, generally before 20 months of age. Prepubertal monkeys exhibit a low frequency and amplitude of LH pulses and there is little nocturnal increase in LH (5). The “early pubertal stage” is defined as the time between the appearance of the first external signs of puberty and menarche. The first external signs of female puberty, such as a slight increase in the nipple size and subsequent swelling of perineal sex-skin, usually occur at 20–25 months of age. These external signs of puberty are a consequence of increased levels of circulating gonadotropins and ovarian estrogens: The LH pulse amplitude starts to increase and a nocturnal elevation of gonadotropin levels becomes prominent (5). Subsequently, menarche occurs at 26–30 months of age. After menarche, females have irregular menstrual cycles without ovulation. Mean LH levels, LH pulse amplitude (not pulse frequency), and nocturnal LH further increase and at 36–45 months of age, monkeys start to ovulate. We have defined this developmental stage between menarche and first ovulation as the “midpubertal stage” [Figure 1; (5)].

## Release of GnRH and Kisspeptin Increases at Puberty

### GnRH Release

An increase in GnRH is a prerequisite for the initiation of puberty. This concept is based on an experiment showing that pulsatile infusion of GnRH in sexually immature female monkeys by infusion pump resulted in precocious puberty (6) and that an increase in GnRH release occurs at puberty onset in female rhesus monkeys (7). In the prepubertal female, GnRH release is pulsatile and characterized by low mean levels, low pulse frequency, low amplitude, and no nocturnal increases (7). In early pubertal females, mean GnRH levels, pulse frequency, and pulse amplitude are all increased, and nocturnal increases in GnRH release start to appear (7, 8). In midpubertal females, mean GnRH levels and pulse-amplitude, but not pulse frequency, further increase reaching the highest levels (7). Additionally, nocturnal GnRH increases become most prominent (7, 8). A similar pubertal increase in pulsatle GnRH release in rodents and sheep (9–11) has been shown by direct measurements, and in humans by indirect LH measurements (12–14). Because the pubertal increase in GnRH release is ovarian steroid independent (1), ovariectomized (OVX) females at the prepubertal stage exhibit a low mean, low pulse frequency, and low pulse amplitude GnRH release, similar to those in gonadally intact counterparts. In OVX females, at the early and midpubertal stages, mean GnRH levels and GnRH pulse amplitude are much higher than in ovarian intact females, but the pulse frequency stays similar, at ~1 pulse/h (8). A similar pubertal change in LH release in human gonadal dysgenesis patients with Turner’s syndrome has also been reported (14, 15).

### Kisspeptin Release

As we described for GnRH release, kisspeptin is released in the median eminence in a pulsatile manner (16). Additionally, kisspeptin release in females undergoes pubertal changes, parallel to those with GnRH release. The mean release, pulse frequency, and pulse amplitude of kisspeptin release in pubertal female monkeys are all higher than those in prepubertal females (17). Again, examination of the effects of OVX on kisspeptin release indicates that while OVX stimulates kisspeptin release in pubertal females, it does not change in prepubertal females (17). That is, kisspepetin release in prepubertal OVX females is characterized with low mean release, low pulse frequency, and low amplitude similar to those in ovarian intact prepubertal females, whereas kisspeptin release in pubertal OVX females consists of higher mean release and higher pulse amplitude, but not higher pulse frequency, when compared to ovarian intact pubertal females (17). Therefore, the pubertal increase in kisspeptin release in primates is ovarian steroid independent. Importantly, however, similar to GnRH release (18), treatment with estradiol suppresses elevated kisspeptin levels in pubertal females, whereas estradiol does not change kisspeptin levels in prepubertal females (17).

In humans, elevated levels of circulating kisspeptin in association with precocious puberty or premature thelarche have been reported (19–23). This is consistent with our results derived from direct kisspeptin measurements in the hypothalamus. Nevertheless, the validity of the finding in human studies is unclear, as circulating kisspeptin may not be of hypothalamic origin. In mammalian species, kisspeptin is synthesized not only in the various part of the brain (24) and placenta but also in peripheral tissues such as the adrenals, ovaries, testes, and kidney (25–28).

## GnRH Response to the Kisspeptin Receptor Agonist, Kisspeptin-10, Increases at Puberty

Since its discovery, kisspeptin has been identified as the most powerful secretagoue for GnRH release (29). GnRH neurons express kisspeptin receptors (Kiss1r) (30, 31), kisspeptin-10 (hKP10) directly depolarizes GnRH neurons and sensitivity of GnRH response to kisspeptin undergoes pubertal changes in rodents (30). In humans and monkeys, contacts between GnRH and kisspeptin neuroterminals in the median eminence, which is indicative of a non-synaptic signaling mechanism, have been reported (32, 33).

**Figure 2:**
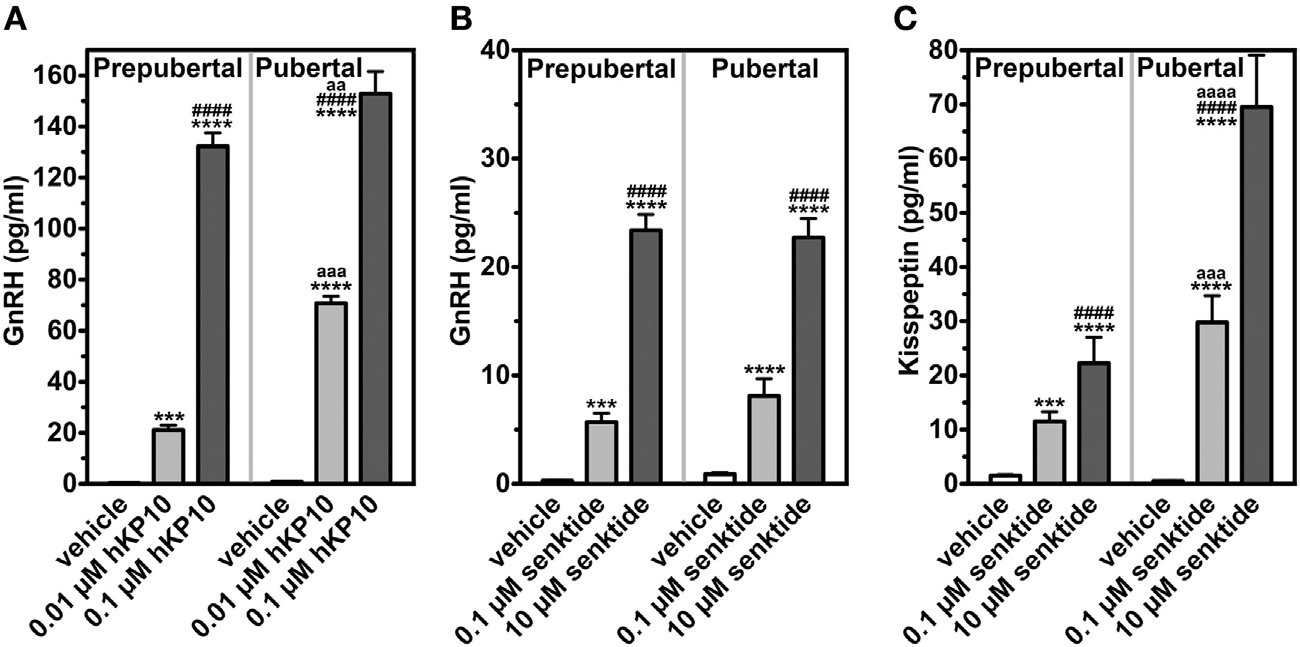
Changes in release of GnRH and kisspeptin (area under the curve in response to challenge of secretagogues). GnRH in response to human kisspeptin-10 (hKP10) **(A)** and senktide **(B)** in female rhesus monkeys are shown. Kisspeptin response to senktide **(C)** is also shown. ***p < 0.001; ****p < 0.0001 vs. vehicle control (within a group). ^####^*p* < 0.0001 vs. lower dose (within a group). aa: *p* < 0.01; aaa: *p* < 0.001; aaaa: *p* < 0.0001 vs. prepubertal stage (between groups at the same dose of challenge). Modified from Ref. (35) with data from Ref. (34) with Copyright Permission.

To clarify the role of kisspeptin signaling in the pubertal increase in GnRH in female monkeys, we first assessed the manner in which the GnRH response to hKP10 changes throughout puberty. GnRH neurons in gonadally intact prepubertal and pubertal females respond to human hKP10 at 0.01 and 0.1 µM doses in a dose-responsive manner (34). Importantly, the GnRH response to hKP10 at the same dose in pubertal females is larger than that in prepubertal females [Figure 2; (34)]. This indicates that GnRH neurons in pubertal monkeys are more sensitive than in prepubertal monkeys.

Because circulating gonadal steroid levels between prepubertal and pubertal animals differ, higher sensitivity of GnRH neurons to kisspeptin signaling in pubertal females may be due to circulating steroids, namely estradiol. Accordingly, we examined the effect of OVX on the developmental changes in GnRH responses to hKP10 in female monkeys (34). While OVX in prepubertal animals does not alter GnRH response to hKP10, OVX completely abolished the hKP10-induced GnRH release in OVX pubertal females. Importantly, estradiol replacement in OVX pubertal females only partially restores the hKP10-induced GnRH release, suggesting that circulating estradiol is important for kisspeptin action on GnRH neurons in pubertal females (34). One can argue that the absence of GnRH response to hKP10 in OVX monkeys is due to the limitation of the maximized kisspeptin neurosecretory capacity after OVX. We believe this is not the case, because (1) two doses (10 and 100 nM) of hKP10 failed to stimulate GnRH release in pubertal OVX females, whereas in prepubertal females, the lower dose (10 nM) is sufficient to stimulate GnRH release (34), and (2) hKP10 (10 nM) can stimulate GnRH release in OVX pubertal females after priming with estradiol (34), although GnRH release in estradiol primed OVX pubertal females was much smaller than that in ovarian intact pubertal females (34). Therefore, it is likely that once KISS1R is exposed to estradiol after the onset of puberty, the properties of KISS1R are altered, such that normal KISS1R function requires the presence of circulating estradiol or, at least, a periodical exposure to estradiol. This speculation, however, needs to be experimentally confirmed by examining whether changes in the KISS1R properties occur in the presence or absence of estradiol and how developmental factors are involved in the mechanism of the estrogen-induced KISS1R property change.

Collectively, we can interpret our findings to mean that the contribution of kisspeptin signaling to the pubertal increase in GnRH release in female monkeys is twofold: first, after puberty onset, a larger amount of kisspeptin is available to stimulate GnRH release, and second, sensitivity of KISS1R on GnRH neurons is higher because of the pubertal increase in circulating estradiol.

## GnRH Response to the NKB Agonist, Senktide, Does Not Undergo Pubertal Change

Neurokinin B action is primarily mediated by NK3R encoded by the *TACR3* gene. Whether GnRH neurons express NK3R is somewhat controversial. While direct application of the NK3R agonist senktide on sliced brain preparation stimulates GnRH neuronal activity in mice (36) and the NK3R is described in close proximity to GnRH neuroterminals in rats and sheep (37, 38), only a small number or no GnRH neuronal cell bodies express NK3R in rat and mice (37, 39, 40). In mice and sheep, however, kisspeptin, NKB, and dynorphin (KNDy) neurons expressing NK3R in the arcuate nucleus (ARC) appear to mediate NKB action to GnRH neurons (39–41). Importantly, however, NKB neurons can signal to GnRH neurons directly at the median eminence, as similar to GnRH fibers, abundant NKB fibers project into the median eminence and GnRH neuroterminal fibers readily express NK3R in the median eminence of rats and humans (33, 37, 42, 43).

We examined the effects of the NKB agonist, senktide, on GnRH release in gonadally intact prepubertal and pubertal female monkeys. Senktide infusion into the median eminence at 0.1 and 10 µM stimulated GnRH release in a dose responsive manner within the same developmental stage [(35); Figure 2]. However, neither 0.1 nor 10 µM senktide results in developmental amplification. The results indicate that the NKB system appears not to be sensitive to the pubertal increases in steroid hormones. We have not conducted the parallel experiments in OVX monkeys.

Stimulatory effects of senktide on GnRH release in our study are consistent with those reported in juvenile orchidectomized male rhesus monkeys assessed by LH measurement (44). However, in rodents, both stimulatory and inhibitory effects of NKB on LH/GnRH release (depending on sex, gonadal status, and ages) have been reported (39, 40, 45–47).

## Kisspeptin Response to the NKB Agonist, Senktide, Undergoes Pubertal Change

Kisspeptin, NKB, and dynorphin (KNDy) are 100% co-localized in the ARC and express estrogen receptor alpha in sheep. Based on the anatomical characteristics along with the self-regulating stimulatory and inhibitory circuitry between NKB, kisspeptin, and dynorphin, Goodman and co-workers (48, 49) have proposed the hypothesis that KNDy neurons in the ARC are responsible for GnRH pulse-generation (50). Subsequently, this concept, including the 100% colocalization rate of three peptides in the ARC, and KNDy neurons as a driver of GnRH pulse-generation, has been confirmed in several species, including rats, mice, and goats (51–54). Nevertheless, we have hypothesized that kisspeptin, NKB, and dynorphin neurons in the hypothalamus of monkeys form a network as each independent unit. This hypothesis is based on the reports that (1) in the human hypothalamus the co-localization rate of kisspeptin, NKB, and dynorphin in the infundibular nucleus (aka ARC) is considerably lower than in other species (33, 55), (2) co-localization of kisspeptin and NKB fibers in the median eminence in humans is relatively rare (33) although this is not the case in male monkeys (56), and (3) unlike in rodents (57, 58), perikarya of kisspeptin neurons in monkeys and human and perikarya of NKB neurons in humans are seen in the median eminence (32, 33).

As the first step to test this hypothesis, we measured kisspeptin in the same samples collected from the median eminence, in which the effects of senktide on GnRH release were examined. The effects of senktide on kisspeptin release in females are strikingly parallel to its effects on GnRH (35). Kisspeptin responses to senktide at 0.1 and 10 µM in females are dose dependent within the developmental stage. However, senktide at both 0.1 and 10 µM doses yield an approximately twofold developmental amplification of kisspeptin release in females [(35); Figure 2]. We speculate that circulating estradiol is responsible for the developmental amplification of senktide-induced kisspeptin release, as the female kisspeptin system is highly sensitive to estradiol.

The important question here is why a larger release of kisspeptin induced by senktide in pubertal females than prepubertal females is not directly transduced to a larger GnRH release? We speculate that this is due to involvement of opioid input, as opioid tone increases after puberty onset. In fact, it has been shown that opioid tone increases along with the pubertal increase in estradiol/testosterone. For example, while administration of antagonists for opioid peptides, such as naloxone and naltrexone, in prepubertal children, chimpanzees, and rhesus monkeys failed to stimulate LH/GnRH release (59–64), these opioid antagonists consistently suppress pulsatile LH release in sexually mature humans and monkeys (65–68). Moreover, proopiomelanocortin mRNA expression increases along with progress of puberty in male monkeys (69) and β-endorphin release in the median eminence increases in association with puberty onset in female monkeys (70). A similar view has been reported in ewe (71). Perhaps, the pubertal increase in stimulatory kisspeptin and NKB signaling tones is counterbalanced by opioid peptides. Additional investigations are needed to confirm this view.

## Developmental Changes in the Neurocircuits Involved in the Pubertal Increase in GnRH Release

**Figure 3:**
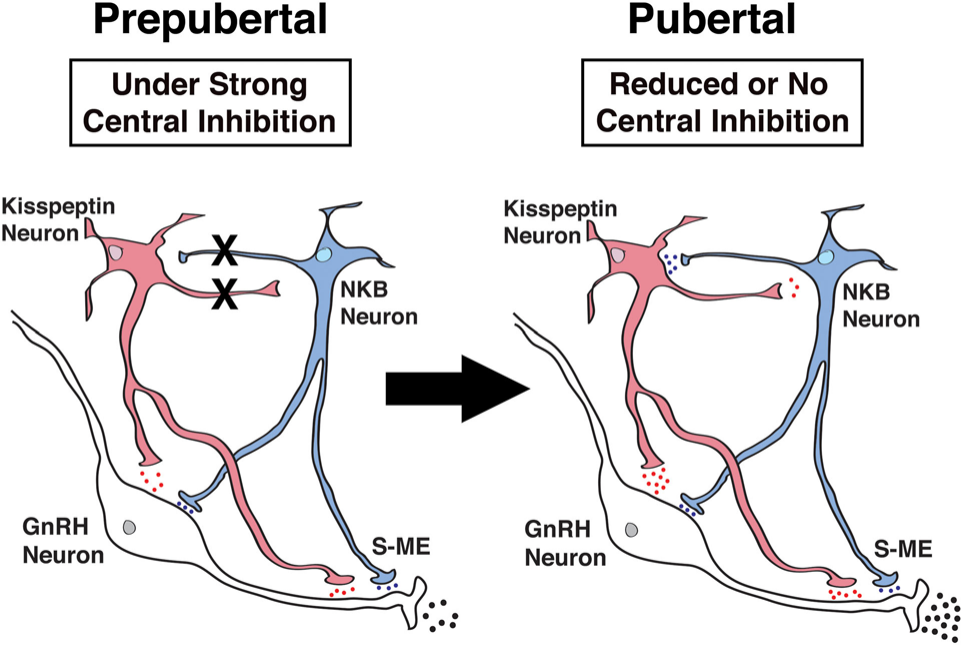
Schematic diagram showing the developmental changes in kisspeptin (red) and neurokinin B (NKB) (blue) signaling to GnRH neurons in the stalk-median eminence (S-ME) in prepubertal (*left panel*) and pubertal (*right panel*) female monkeys. Note that X’s between kisspeptin and NKB neurons indicate the absence of signaling pathways and the blue, red and black dots indicate relative amount of neuropeptide release. Kisspeptin signaling to NKB neurons is hypothetical, as in the present study, we did not measure the kisspeptin-induced NKB release. Modified from Ref. (35) with Copyright Permission.

As described above, both hKP10 and senktide stimulate GnRH release in a dose-responsive manner in prepubertal as well as pubertal female monkeys (34, 35). We also described that senktide greatly stimulates kisspeptin release in a dose-dependent manner in both prepubertal and pubertal females (35). However, these observations in females do not suggest any hierarchical relationship between NKB and kisspeptin signaling. Moreover, the network between kisspeptin and NKB signaling may undergo pubertal changes. Therefore, in the next series of studies, we have examined whether NKB signaling is mediated through kisspeptin neurons or kisspeptin signaling is mediated through NKB neurons using respective agonists and antagonists. The results indicate that the senktide-induced GnRH release is blocked in the presence of the KISS1R antagonist, peptide 234, in pubertal, but not prepubertal monkeys (35). Similarly, hKP10-induced GnRH release is blocked by the NK3R antagonist SB222200 in pubertal, but not prepubertal monkeys (35). These results suggest that while in prepubertal female monkeys, kisspeptin and NKB signaling influences GnRH release as independent units, in pubertal female monkeys, a reciprocal signaling network (i.e., NKB signaling through kisspeptin neurons and kisspeptin signaling through NKB neurons) is established (Figure 3). This cooperative mechanism by the kisspeptin and NKB networks appears to underlie the pubertal increase in GnRH release in female monkeys. We speculate that the cooperative mechanism between kisspeptin and NKB signaling to GnRH release would ensure the success of complex reproductive functions in females.

Our findings reported in this manuscript are obtained from dialysates collected from the median eminence, where agonists and antagonists for kisspeptin and NKB are directly infused. Because of our technical precision (72), interactions between GnRH and kisspeptin neurons, kisspeptin and NKB neurons, and GnRH and NKB neurons are likely taking place at the median eminence and infundibular stalk (extended median eminence). As we discussed above, the primate median eminence appears to be equipped for this purpose. In the median eminence neuroterminal interactions between NKB, kissepeptin, and GnRH neurons are likely to occur through a non-synaptic mechanism, but the presence of kisspeptin, NKB, and GnRH neuronal cell bodies in the median eminence (32, 33, 73, 74) indicates possible synaptic interactions as well. Nevertheless, currently, we do not know the degree to which the median eminence kisspeptin-NKB system is influenced by the infundibular (ARC) kisspeptin-NKB system. It will be a major task to clarify the mechanisms of developmental changes in these signaling pathways that regulate GnRH release.

## Pulsatility of GnRH Release and Timing of Puberty

Genetic findings in humans indicate that both kisspeptin and NKB signaling is critical for the mechanism governing puberty onset (2–4). Similar findings in kisspeptin and NKB knockout mice were also reported (75, 76). As we discussed above, kisspeptin signaling itself (77, 78) or the KNDy network (50) is indispensable for pulsatility of GnRH release and an increase in pulsatile GnRH release is required for puberty onset (1). Here, a critical question arises as to whether kisspeptin and NKB signaling determines the timing of puberty in primates. In other words, does an increased activity of kisspeptin signaling/KNDy network during development facilitate pulsatile GnRH release initiating puberty onset OR is an increased activity of kisspeptin signaling/KNDy network a consequence of reduction in “Central Inhibition”? The following is our view.

In primates, GnRH neurons in the hypothalamus are already active at birth and elevated GnRH neuronal activity induces a so-called “mini-puberty” during the neonatal period (79). However, activity of the GnRH neurosecretory system is suppressed by “Central Inhibition” and becomes dormant throughout the prepubertal period (80). Neuronal substrates that represent “Central Inhibition” are currently unclear. Our previous studies indicate that tonic inhibition by γ-aminobutyric acid (GABA) neurons may be one component (1, 81) and neuroestradiol (72) might be another component. It has also been postulated that MKRN3 protein may be responsible for suppression of GnRH release before puberty, as mutations of the *makorin RING finger protein 3* gene (*MKRN3*) result in precocious puberty in humans (82). More recently, based on the gene array comparison between castrated prepubertal and pubertal male monkey hypothalami, followed by physiological experiments, the transcriptional repressor protein, GATAD1, is postulated as a substrate responsible for prepubertal GnRH suppression (83). Nevertheless, the report that the kisspeptin antagonist, peptide 234, blocks the GABA_A_ antagonist bicuculline-induced GnRH increase in prepubertal females (84) suggests that “Central Inhibition” by GABA is upstream of the kisspeptin signaling system. We speculate that GABA is also upstream of NKB signaling and the NKB antagonist SB222200 would block the GABA_A_ antagonist bicuculline-induced GnRH increase in prepubertal females. Therefore, removal or reduction in “Central Inhibition” is a prerequisite for allowing the pubertal increase in activity of kisspeptin neurons or the KNDy network (Figure 3). Once kisspeptin/KNDy neurons become active, kisspeptin and NKB signaling ensures the pulsatile GnRH release, resulting in the onset of puberty.

The concept of “Central Inhibition” is well documented in humans (85) and rhesus monkeys (1, 86), but it remains controversial in non-primate species. In fact, there are several species differences in the mechanism of puberty onset: (1) As described above, while neonatal castration in primates induces elevated LH/FSH release only transiently (87), the same procedure in rats and sheep results in a sustained increase in gonadotropin release throughout life (11, 88); (2) while the GnRH neuroscretory system in prepubertal monkeys is insensitive to estradiol and sensitivity to estradiol negative feedback is acquired during the early pubertal stage (18), the GnRH neurosecretory system in rodents is highly sensitive to estradiol action during the entire juvenile period and sensitivity to estradiol decreases after first ovulation (88); and (3) while precocious puberty induced by infusion of pulsatile GnRH or *N*-methyl-d-aspartic acid (NMDA) in prepubertal monkeys is halted by the cessation of the infusion (6, 89), precocious puberty induced in rodents with a similar treatment, such as NMDA administration, leads to the maintenance of adult gonadal function after discontinuation of treatment, i.e., NMDA-induced precocious puberty in rats is followed by cyclic ovulation (90).

Despite these species differences, however, in rodents, there are some parallel findings consistent with the concept of the “Central Inhibition” described in primates. For example, in mice, Mkrn3 mRNA expression in the ARC is highest during the first 10–12 postnatal days (P), starts to decrease at P15, and becomes the lowest by P30, just prior to vaginal opening (82), and overexpression of human *GATAD1* gene by transfection in the mouse ARC results in delayed puberty, as postulated in prepubertal monkeys (83). Collectively, it appears that “Central Inhibition” is present in the rodent brain, but its functional significance may differ from that in primates.

## Conclusion

We have shown that both kisspeptin signaling and NKB signaling appear to contribute to the pubertal increase in GnRH release independently or in concert in females. That is, while there is no interaction between kisspeptin and NKB signaling in sexually immature females, increases in kisspeptin signaling through NKB neurons and NKB signaling through kisspeptin neurons both augment the pubertal increase in GnRH release during the progress of puberty. The contribution of direct NKB signaling to GnRH release, however, may be secondary, as NKB signaling to GnRH release does not change across puberty, whereas NKB signaling to kisspeptin release greatly increases (Figure 2). Thus, in females, kisspeptin signaling appears to be the main force driving the pubertal GnRH release increases with their signaling intensity and an increased sensitivity of the receptor, KISS1R [(17, 34); Figure 2]. The role of NKB in the pubertal increase in GnRH release, however, requires further experiments, measuring developmental changes in NKB release in the presence or absence of kisspeptin agonists/antagonists.

We speculate that, in females, reciprocal signaling pathways between kisspeptin and NKB neurons would provide efficiency and flexibility for the stimulation of GnRH release, which ensures complex reproductive functions, such as cyclic ovulations and pregnancy. In summary, kisspeptin signaling and NKB signaling are both indispensable to facilitate the pubertal increase in GnRH after removal or diminution of “Central Inhibition.” Further studies, such as measurements of NKB release in the hypothalamus and examination of the role of dynorphin would strengthen our views.

## Author Contributions

ET and JPG designed experiments. JPG and KLK conducted experiments. JPG analyzed the data, and ET, JPG, and SBS wrote the manuscript.

## Conflict of Interest Statement

The authors declare that the research was conducted in the absence of any commercial or financial relationships that could be construed as a potential conflict of interest. The reviewer VN declared a shared affiliation, with no collaboration, with one of the authors, SBS, to the handling Editor.
